# Bioactive Steroids with Structural Diversity from the South China Sea Soft Coral *Lobophytum* sp. and Sponge *Xestospongia* sp.

**DOI:** 10.3390/md23010036

**Published:** 2025-01-13

**Authors:** Lin-Mao Ke, Zi-Ru Zhang, Song-Wei Li, Yan-Bo Zeng, Ming-Zhi Su, Yue-Wei Guo

**Affiliations:** 1 Shandong Laboratory of Yantai Drug Discovery, Bohai Rim Advanced Research Institute for Drug Discovery, Yantai 264117, China; klm102198@163.com (L.-M.K.); 18789783359@139.com (Z.-R.Z.); 2 Hainan Provincial Key Laboratory for Functional Components Research and Utilization of Marine Bio-Resources, Institute of Tropical Bioscience and Biotechnology, Chinese Academy of Tropical Agricultural Sciences, Haikou 571101, China; 3 Ocean College of Hebei Agricultural University, Qinhuangdao 066000, China; 4 School of Medicine, Shanghai University, Shanghai 200444, China; songweili@shu.edu.cn

**Keywords:** soft coral, sponge, oxygenated steroids, antibacterial activity, anti-inflammatory activity

## Abstract

A chemical investigation of the soft coral *Lobophytum* sp. and the sponge *Xestospongia* sp. from the South China Sea led to the isolation of five steroids, including two new compounds (**1** and **4**) and one known natural product (**3**). Compounds **1**–**3** were derived from the soft coral *Lobophytum* sp., while **4** and **5** were obtained from the sponge *Xestospongia* sp. The structures of these compounds were determined by extensive spectroscopic analysis, the time-dependent density functional theory–electronic circular dichroism (TDDFT-ECD) calculation method, and comparison with the spectral data previously reported in the literature. The antibacterial and anti-inflammatory activities of isolated compounds were evaluated in vitro. Compounds **1**–**3**, **4**, and **5** exhibited weak antibacterial activity against vancomycin-resistant *Enterococcus faecium* G1, *Streptococcus parauberis* KSP28, *Photobacterium damselae* FP2244, *Lactococcus garvieae* FP5245, and *Pseudomonas aeruginosa* ZJ028. Moreover, compound **3** showed significant anti-inflammatory activity by inhibiting lipopolysaccharide (LPS)-induced NO production in RAW 264.7 cells, with an IC_50_ value of 13.48 μM.

## 1. Introduction

The prospect of a post-antibiotic era has become a pressing and dangerous reality due to the rapid global spread of multidrug-resistant bacteria. This alarming situation underscores the urgent need to discover and develop new antibiotics from natural sources. Soft corals of the genus *Lobophytum* (phyulum Cnidaria, class Anthozoa, subclass Octocorallia, order Alcyonacea, family Alcyoniidae) and sponges of the genus *Xestospongia* (phyulum Porifera, class Demospongia, order Haplosclerida, family Petrosiidae) are widely distributed tropical animals that have been chemically investigated since the 1970s, and are well known for their rich variety of complex secondary metabolites [1,2]. Among these various classes of secondary metabolites, steroids are among the characteristic compounds produced by these animals, which reportedly exhibit various of significant pharmacological activities, including antimicrobial, anti-tumor, and anti-inflammatory effects [3]. Therefore, the chemical diversity and excellent biological activities of steroids have long been of great interest to marine natural product chemists and pharmacologists, as they are potential agents for marine drug development [4,5,6,7,8].

As part of our ongoing search for bioactive steroids from marine invertebrates, we have recently collected the soft coral *Lobophytum* sp. from Xisha Island, Hainan province, China, and sponge *Xestospongia* sp. from Xuwen Country, Guangdong Province, China, respectively. The chemical investigations of the Et_2_O-soluble fraction from the acetone extract of *Lobophytum* sp. have led to the isolation of previously undescribed polyoxygenated steroid **1** and known compounds **2** and **3**, and the fractionation of *Xestospongia* sp. extract resulted in the isolation of a previously undescribed polyoxygenated steroid **4** together with known compound **5.** Herein, we report the isolation, structural determination, and bioactivity evaluation of these compounds.

## 2. Results

**Figure 1:**
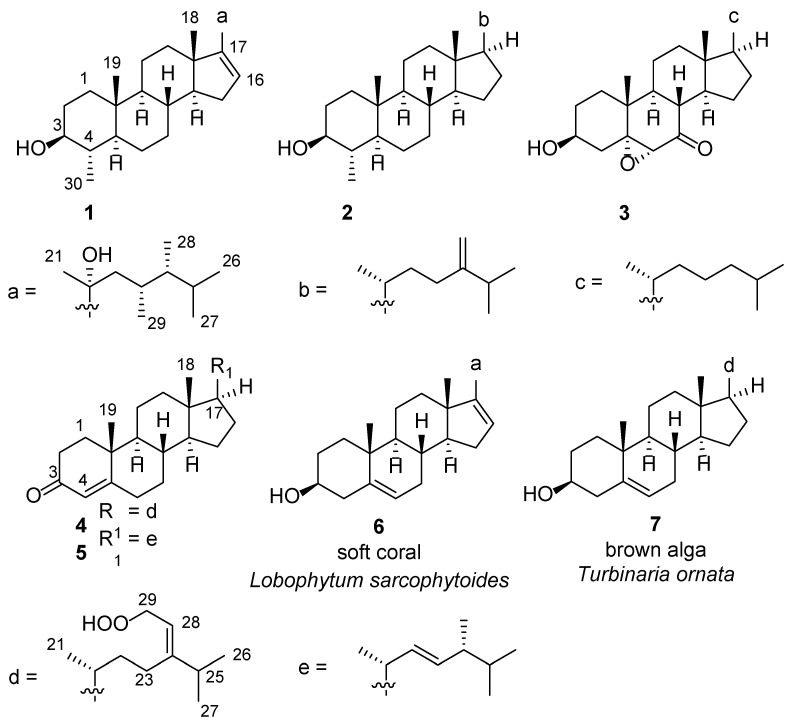
Chemical structures of compounds **1**–**7**.

Samples of *Lobophytum* sp. and *Xestospongia* sp. were cut into pieces and exhaustively extracted with acetone. The Et_2_O-soluble portions of the acetone extracts were subjected to repeated column chromatography and yielded three steroids (**1**–**3**) from *Lobophytum* sp. and two steroids (**4** and **5**) from *Xestospongia* sp., respectively. Steroids **2**, **3**, and **5** were readily identified as (3*β*,4*α*,5*α*)-4-methylergost-24(28)-en-3-ol [9], (3*β*,5*α*,6*α*)-5,6-epoxy-3-hydroxycholestan-7-one [10] and (22*E*,24*S*)-ergosta-4,22-dien-3-one [11], respectively, via a comparison of their NMR data and optical rotation values with those reported in the literature (Figure 1).

**Figure 2:**
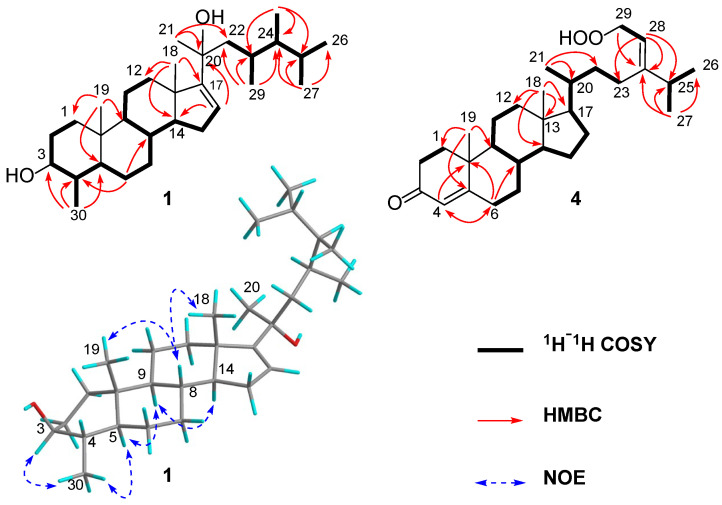
^1^H–^1^H COSY, HMBC correlations of **1** and **4**, and key NOE correlations of **1**.

Compound **1** was obtained as a white powder. Its molecular formula was determined as C_30_H_52_O_2_ due to a HR-ESIMS protonated molecule ion peak at *m*/*z* 445.4034 [M + H]^+^ (caled. for C_30_H_53_O_2_, 445.4040), which was appropriate for five degrees of unsaturation. The IR spectrum of **1** showed a characteristic absorption band for hydroxyl group (3414 cm^−1^). The ^1^H NMR data of compound **1** (Table 1) showed a trisubstituted double bond at *δ*_H_ 5.49 (1H, br d, *J* = 1.8 Hz), an oxymethine group at *δ*_H_ 3.10 (1H, m), and eight methyls at *δ*_H_ 0.98 (3H, s), 0.88 (3H, s), 1.36 (3H, s), 0.91 (3H, d, *J* = 6.6 Hz), 0.89 (3H, d, *J* = 5.8 Hz), 0.78 (3H, d, *J* = 6.8 Hz), 0.80 (3H, d, *J* = 6.9 Hz), and 0.97 (3H, d, *J* = 5.6 Hz). Furthermore, the ^13^C NMR data (Table 1) and DEPT spectrum of compound **1** indicated the presence of thirty signals, which consisted of eight methyls, eight methylenes, ten methines (including an olefinic at *δ*_C_ 124.0, an oxymethine at *δ*_C_ 76.6, and sp^3^ hybridized at *δ*_C_ 29.8, 31.0, 33.6, 39.4, 45.6, 51.3, 54.9, 58.1), and four quaternary carbons (including an oxygenated carbon at *δ*_C_ 76.0, an olefinic at *δ*_C_ 161.1, and two sp^3^ hybridized at *δ*_C_ 36.3, 47.7). The abovementioned 1D NMR data suggest the presence of a double bond, which accounted for one degree of unsaturation. Thus, the four remaining degrees of unsaturation were ascribed to a tetracyclic system in **1**. A comparison of the 1D NMR data of **1** with those of a co-occurring 4*α*-methyl steroid, (3*β*,4*α*,5*α*)-4-methylergost-24(28)-en-3-ol (**2**), revealed that they were structural analogues, with the major difference being the structure of the side chains, which was further confirmed by a detailed analysis of the cross-peaks in its ^1^H−^1^H COSY and HMBC spectra. In detail, an analysis of ^1^H−^1^H COSY relationships provided two sequential structure fragments. Finally, with the aid of the key HMBC correlations from Me-19 to C-1, C-5 and C-10; from Me-18 to C-12, C-14 and C-17; from Me-21 to C-20 and C-22; from Me-27 to C-24, C-25 and C-26; from Me-28 to C-23, C-24 and C-25; from Me-29 to C-22, C-23 and C-24; and from Me-30 to C-3, C-4 and C-5, we assigned the planar structure of **1** (Figure 2).

The relative configuration of **1** was established mainly via analysis of the NOESY data (Figure 2). The correlations of H-3 (*δ*_H_ 3.10)/H_3_-30 (*δ*_H_ 0.97), H_3_-30/H-5 (*δ*_H_ 0.77), H-5/H-9 (*δ*_H_ 0.71), and H-9/H-14 (*δ*_H_ 1.39) revealed that these protons were disposed at the same side of the molecule and randomly assigned as *α*-oriented. Subsequently, the interactions of H_3_-18 (*δ*_H_ 0.98)/H-8 (*δ*_H_ 1.56) and H_3_-19 (*δ*_H_ 0.88)/H-8 revealed that these protons and/or proton-bearing groups were positioned at the other side of the molecule, and thus were *β*-directed. Considering the high agreement in ^13^C NMR spectroscopic data of **1** and **2**, the same 3S*,4S*,5S*,8R*,9S*,10R*,13S*,14S* configurations were suggested. Furthermore, the relative configuration of chiral carbons C-20, 23, and 24 was tentatively assigned by the comparison of NMR data with those of model compound sarcophytosterol (**6**), a previously reported steroid isolated from the Dongsha atoll soft coral *Lobophytum sarcophytoides*, whose absolute configuration was determined by single-crystal X-ray diffraction analysis [12]. The absolute configuration of **1** was determined based on the consideration of the biosynthetic pathway of steroid compounds. Finally, the structure of compound **1** was thus elucidated as (3*S*,4*S*,5*S*,20*R*,23*R*,24*R*)-4,23-dimethylergost-3,20-diol.

Compound **4** was obtained as a white powder. Its molecular formula was determined as C_29_H_46_O_3_ by an HR-ESIMS ion peak at *m*/*z* 443.3520 [M + H]^+^ (caled. for C_29_H_47_O_3_, 443.3520), appropriate for seven degrees of unsaturation. The IR spectrum of **4** showed characteristic absorption bands for hydroxy (3414 cm^−1^) and conjugated carbonyl (1674 cm^−1^). The ^1^H NMR data of compound **4** (Table 1) showed the general characters of sterols, including two olefinic protons resonating at *δ*_H_ 5.72 (1H, s, H-4) and 5.32 (1H, t, *J* = 7.3 Hz, H-28), an oxymethylene at 4.55 (2H, d, *J* = 7.3 Hz, H_2_-29), and five methyls at *δ*_H_ 0.72 (3H, s, Me-18), 1.18 (3H, s, Me-19), 0.98 (3H, d, *J* = 6.6 Hz, Me-21), 1.03 (3H, d, *J* = 6.8 Hz, Me-26), and 1.04 (3H, d, *J* = 6.8 Hz, Me-27). Furthermore, the ^13^C NMR data (Table 1) and DEPT spectrum of compound **4** indicated the presence of twenty-nine carbons, which consisted of five methyls, eleven methylenes, eight methines (including two olefinics at *δ*_C_ 115.1, 123.9 and six sp^3^ hybridized at *δ*_C_ 36.3, 53.9, 56.0, 55.7, 36.5, 34.8), and five quaternary carbons (including a carbonyl group at *δ*_C_ 199.8, two olefinics at *δ*_C_ 171.8, 155.4, and two sp^3^ hybridized at *δ*_C_ 38.8 and 42.6). The abovementioned data suggest the presence of two double bonds and a carbonyl group, which accounted for three degrees of unsaturation. Thus, the remaining four degrees of unsaturation were ascribed to a tetracyclic system in molecule **4**.

Moreover, ^1^H–^1^H COSY correlations of H_2_-1/H_2_-2, H_2_-6/H_2_-7/H-8/H-9/H_2_-11/H_2_-12, H-8/H-14/H_2_-15/H_2_-16/H-17/H-21/H_3_-20, H-21/H_2_-22/H_2_-23, H_3_-26/H-25/H_3_-27, and H-28/H_2_-29, together with the key HMBC correlations of Me-19 to C-1, C-5, C-9 and C-10, of Me-18 to C-12, C-13, C-14 and C-17; of H-6 to C-4, C-8 and C-10; of Me-21 to C-20 and C-22, of Me-27 to C-24, C-25 and C-26; and of H-28 to C-23, C-24 and C-25 permitted the establishment of the carbon skeleton of stigmastane-type steroid (Figure 2). A comparison of the NMR data of **4** with those of co-isolated compound **5** and model compound (3*β*,24*E*)-29-hydroperoxystigmasta-5,24(28)-dien-3-ol (**7**), a known compound isolated from the Formosan brown alga *Turbinaria ornate* [13], revealed a high degree of similarity in their data (Table 2), indicating that they share the same structural fragments, which further determined the stereo-configuration of compound **4**.

**Figure 3:**
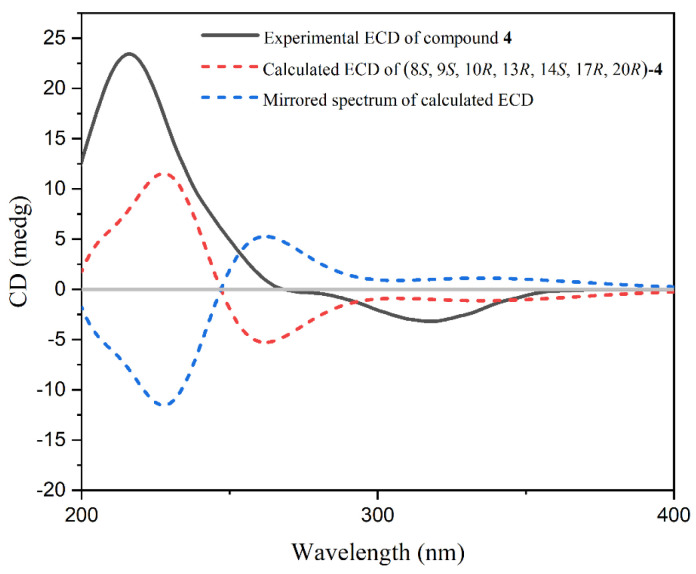
The determination of absolute configuration of **4** by the TDDFT-ECD calculation: experimental ECD spectrum of **4** (black solid line), calculated ECD spectrum of (8*S*,9*S*,10*R*,13*R*,14*S*,17*R*,20*R*)-**4** (red dashed line), and mirrored curve of calculated ECD (blue dashed line).

The presence of the conjugated group in the structure of **4** resulted in the significant Cotton effect in its ECD spectrum. The absolute configuration of **4** can be determined by employing the time-dependent density functional theory–electronic circular dichroism (TDDFT-ECD) calculation method. Therefore, TDDFT-ECD calculation has been performed for compound **4**. As shown in Figure 3, the calculated ECD curve of (8*S*,9*S*,10*R*,13*R*,14*S*,17*R*,20*R*)-**4** matched with the experimental ECD spectrum of **4**, whereas the ECD profile of its enantiomer showed a completely opposite curve. Consequently, the absolute configuration of **4** was fully established as 8*S*, 9*S*, 10*R*, 13*R*, 14*S*, 17*R*, and 20*R*, and compound **4** was named (24*E*)-29-hydroperoxystigmasta-4,24(28)-dien-3-one.

In the antibacterial bioassays, compounds **1**, **3**, and **4**, at a concentration of 100 μM, demonstrated growth inhibitory activity against vancomycin-resistant *Enterococcus faecium* G1. Furthermore, compounds **1** and **3** exhibited weak antibacterial activity against the fish pathogenic bacterium *Streptococcus parauberis* KSP28, with an MIC value of 100 μM. They also showed activity against *Photobacterium damselae* FP2244, with MIC values of 50 μM and 100 μM, respectively, and against *Lactococcus garvieae*, with MIC values of 50 μM and 100 μM, respectively. Compound **5** showed weak antibacterial activity against *Pseudomonas aeruginosa* ZJ028 with an MIC value of 100 μM (Table 2). Compound **2** exhibited no antibacterial activity against these bacterial strains at concentrations of 100 μM.

**Figure 4:**
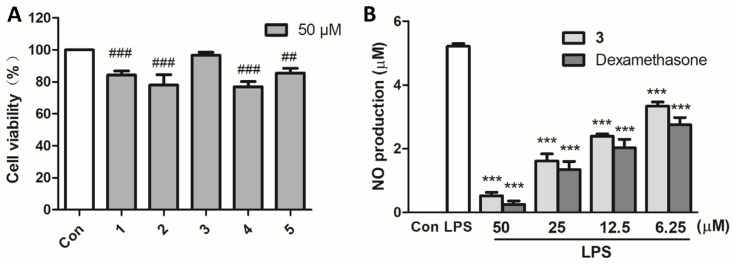
Cell viability and anti-inflammatory activity results. (**A**) Cell viabilities of RAW 264.7 cells treated with isolated compounds for 24 h. (**B**) NO production of RAW 264.7 cells which were pre-treated with **3** or dexamethasone for 1 h, and then stimulated by LPS for 24 h. ^##^ *p* < 0.01 and ^###^ *p* < 0.001 vs. untreated controls; *** *p* < 0.001 vs. LPS-treated cells.

The effect of compounds **1–5** at a concentration of 50 μM on the cell viability of RAW 264.7 cells was evaluated using an MTT assay. The cell viability was 84.3%, 78.0%, 96.6%, 76.9%, and 85.5%, respectively, following treatment with compounds **1**–**5**. Of these, compound **3** exhibited no cytotoxicity toward RAW 264.7 cells at 50 μM (Figure 4A). Consequently, compound **3** was selected for further evaluation in an anti-inflammatory assay. The results revealed that compound **3** exerted dose-dependent anti-inflammatory effects by inhibiting NO production in lipopolysaccharide (LPS)-stimulated RAW 264.7 cells, with an IC_50_ value of 13.48 μM, comparable to that of dexamethasone with an IC_50_ value of 8.36 μM (Figure 4B).

## 3. Discussion

Steroids are an important class of natural products found in a wide range of organisms, including marine soft corals and sponges. This fused-four ring system allows for various structural modifications, such as hydroxyl substitutions, double bonds, epoxidation, and carbonylation, which can lead to compounds with novel and enhanced pharmacological properties. Steroids are commonly associated with anti-inflammatory activity. Dexamethasone and hydrocortisone have been widely used as representative steroid drugs in clinical practice for decades. Researchers have identified many steroids with anti-inflammatory activity derived from soft corals. For instance, polyoxygenated steroids michosterols A-C, featuring three hydroxyl group substitutions, were discovered in the soft coral *Lobophytum michaelae* and have since been used as anti-inflammatory agents [14]. Additionally, lobophysterol E, a steroid with a methyl group at C-4 and a carbonyl substitution at C-6, was isolated from *Lobophytum pauciflorum* and demonstrated a moderate anti-inflammatory effect in a zebrafish model [15]. Two steroids isolated from the soft coral *Sinularia depressa*, characterized by an unusual C-18 oxygenated pattern, exhibited moderate inhibitory activity against LPS-induced TNF-α release, with IC_50_ values of 51.1 μM and 22.7 μM, respectively [16]. In this study, compound 3, which possesses an epoxy group at C-5 and C-6, and a carbonyl group at C-7, demonstrated dose-dependent anti-inflammatory activity with an IC_50_ value of 13.48 μM. Based on a literature review and our research findings, it is suggested that steroids with polyoxygenated substitutions have significant potential for anti-inflammatory activity.

Our group has long been engaged in the discovery of marine-derived steroids, which predominantly exhibit antibacterial activity. We have isolated four new steroids, lobocaloids A–D, from soft coral *Lobophytum catalai* Tixier-Durivault. These compounds exhibited antibacterial activities against the fish pathogenic *S. parauberis* KSP28, with MIC values ranging from 12.3 to 53.6 µg/mL, and exerted moderate inhibitory effects on the pyocyanin production in *Pseudomonas aeruginosa* [17]. In addition, sinulasterol E, an antibacterial steroid isolated from the soft coral *Sinularia depressa*, was acetylated at the C-18 position and features a peroxide group at the C-7 position. It inhibited vancomycin-resistant *E. faecium* with MIC values of 62.5 and 125 μM [18]. Moreover, a series steroid with *α*,*β*-*α*’,*β*’-unsaturated carbonyl moiety in ring A were identified from *Lobophytum* sp. They exhibited significant inhibitory activities against the fish pathogenic bacteria *S. parauberis* FP KSP28, *P. damselae* FP2244, and *S. parauberis* SPOF3K, with IC_90_ values ranging from 0.1 to 11.0 µM, and against the vancomycin-resistant *E. faecium*, with IC_90_ values ranging from 4.4 to 18.3 µM [19]. In this study, we isolated steroids **1** and **3** from soft coral *Lobophytum* sp., and obtained **4** and **5** from sponge *Xestospongia* sp. These compounds also inhibited the growth of fish pathogenic bacteria at a concentration of 50 or 100 µM. We found that steroids with antibacterial activity that were obtained from marine sources exhibit more pronounced activity against halobacteria, including *S. parauberis* FP KSP28, *P. damselae* FP2244, *L. garvieae* FP MP5245, and so on. The presence of antibacterial steroids has been reported extensively in marine organisms, and has been identified not only in soft corals and sponges [20], but also in marine microorganisms [21]. Therefore, we speculate that the antimicrobial steroids found in soft corals and sponges may, to some extent, be derived from their associated microbial communities. These compounds likely served as chemical defense agents that benefit benthic marine animals and help them to protect themselves from microbial invasion in the marine environment.

## 4. Experimental Section

### 4.1. General Methods

NMR spectra were acquired using a Bruker 600 spectrometer (Bruker Biospin AG, Fällanden, Germany). Chemical shifts were reported with the residual CHCl_3_ (*δ*_H_ 7.26) as the internal standard for ^1^H NMR spectrometry and CDCl_3_ (*δ*_C_ 77.2) for ^13^C NMR spectrometry. IR spectra were recorded on a Nicolet 6770 spectrometer (Thermo Fisher Scientific, Madison, WI, USA). HR-ESIMS spectra were obtained using an Agilent 1290-6545 UHPLC-QTOF mass spectrometer. Commercial silica gel (Qingdao Haiyang Chemical Group Co., Ltd., Qingdao, China, 200–300 and 300–400 mesh) and Sephadex LH-20 gel (Amersham Biosciences, Amersham, UK) were used for column chromatography (CC), and precoated silica gel plates (Yan Tai Zi Fu Chemical Group Co., Yantai, China, G60 F-254) were used for analytical TLC. Reversed-phase (RP) HPLC was performed on an Agilent 1260 series liquid chromatography device equipped with a DAD G1315D detector at 210 and 254 nm. All solvents used for CC and HPLC were of analytical grade (Shanghai Chemical Reagents Co., Ltd., Shanghai, China) and chromatographic grade (Dikma Technologies Inc., Beijing, China), respectively. MTT reagent (from Adamas-beta, Shanghai, China), LPS, dexamethasone, Griess reagent (modified), vancomycin hydrochloride, and tetracycline hydrochloride (from Sigma-Aldrich, Darmstadt, Germany) are commercially available reagents.

### 4.2. Animal Materials

The soft coral *Lobophytum* sp. was collected from Ximao Island (18°14′00″ N, 109°22′11″ E), Hainan province, China, in October 2018, and the sponge *Xestospongia* sp. was collected from Xuwen Country (20°13′21″ N, 109°55′21″ E), Guangdong Province, China, in October 2021. Both marine invertebrates were identified by Prof. X.-B. Li from Hainan University. The voucher samples are listed under registration Nos. 18-XD-3 and Y-21-XW-51, respectively, and are available for inspection at the Shandong Laboratory of Yantai Drug Discovery, Bohai rim Advanced Research Institute for Drug Discovery.

### 4.3. Extraction and Isolation

The frozen samples of *Lobophytum* sp. (350.0 g, dry weight after extraction) were cut into small lumpy objects and extracted thoroughly with acetone at room temperature (5.0 L × 4). The acetone extract was evaporated, leaving behind a brown residue. Subsequently, the brown residue was partitioned between H_2_O and Et_2_O. The Et_2_O solution was concentrated to provide a dark residue (0.758 g). Then, the residue was subjected to gradient silica gel (200–300 mesh) column chromatography (CC) [Et_2_O/petroleum ether (PE) 0→100%] and yielded 21 fractions (Fra. 1–21). Fraction 3 was separated by Sephadex LH-20 CC (PE/CH_2_Cl_2_/MeOH, 2:1:1) into five subfractions. Subfraction 3B4 was purified by semi-preparative RP-HPLC (MeOH/H_2_O, 98:2, 3.0 mL/min) to create compound **3** (1.1 mg, *t*_R_ = 20 min). Fraction 6 was initially separated by Sephadex LH-20 CC (PE/CH_2_Cl_2_/MeOH, 2:1:1), resulting in four subfractions. Subfraction 6D was then purified by semi-preparative RP-HPLC (MeOH/H_2_O, 99:1, 3.0 mL/min) to obtain compound **2** (1.6 mg, *t*_R_ = 43 min). Fraction 13 was separated by Sephadex LH-20 CC (PE/CH_2_Cl_2_/MeOH, 2:1:1) into eight subfractions. Subfraction 13C was purified by semi-preparative RP-HPLC (MeOH/H_2_O, 93:7, 3.0 mL/min) to yield compound **1** (1.3 mg, *t*_R_ = 22 min).

The frozen bodies of *Xestospongia* sp. (395 g, dry weight after extraction) were cut into small pieces and extracted exhaustively with acetone at room temperature (5.0 L × 4). The organic extract was evaporated to create a residue, which was partitioned between H_2_O and Et_2_O. The Et_2_O solution was concentrated to create a brown residue (1.9 g). The obtained residue was subjected to gradient silica gel CC [Et_2_O/PE, 0→100%] and yielded 7 fractions (Fr. 1–7). Fraction 4 was separated by Sephadex LH-20 CC (PE/CH_2_Cl_2_/MeOH, 2:1:1) into six subfractions. Subfraction 4E was purified by semi-preparative RP-HPLC (MeCN/H_2_O, 93:7, 3.0 mL/min) to yield compound **4** (2.4 mg, *t*_R_ = 51 min). Fraction 7 was separated by Sephadex LH-20 CC (PE/CH_2_Cl_2_/MeOH, 2:1:1) into six subfractions. Subfraction 7D3 was purified by semi-preparative RP-HPLC (MeOH/H_2_O, 93:7, 3.0 mL/min) to yield compound **5** (6.3 mg, *t*_R_ = 32 min).

#### 4.3.1. (3*S*,4*S*,5*S*,20*R*,23*R*,24*R*)-4,23-Dimethylergost-3,20-diol (**1**)

White amorphous powder; ${\left[\alpha \right]}_{D}^{20}$ −2.4 (*c* 0.17, MeOH); IR (KBr): *ν*_max_ 3414, 2940, 2849, 1456, 1371, 962 cm^−1^; ^1^H and ^13^C NMR data (see Table 1); HR-ESIMS at *m*/*z* 445.4034 [M + H]^+^, (calcd. for C_30_H_53_O_2_, 445.4040).

#### 4.3.2. (24*E*)-29-Hydroperoxystigmasta-4,24(28)-dien-3-one (**4**)

White amorphous powder; ${\left[\alpha \right]}_{D}^{20}$ +54 (*c* 0.2, MeOH); UV (CH_3_CN) *λ*_max_ (log*ε*) 240 (3.0) nm; ECD (1.1 mM, CH_3_CN) *λ*_max_ (Δ*ε*) 318 (−3), 217 (+24) nm; IR (KBr): *ν*_max_ 3325, 2941, 2860, 1674, 1618, 1377, 1230, 1182 cm^−1^; ^1^H and ^13^C NMR data (see Table 1); HR-ESIMS at *m*/*z* 443.3520 [M + H]^+^, (calcd. for C_29_H_47_O_3_, 443.3520), *m*/*z* 425.3415 [M-H_2_O + H]^+^, (calcd. for C_29_H_45_O_2_, 425.3414).

### 4.4. TDDFT-ECD Calculation

A conformational search for compound **4** was carried out according to the general protocols that were previously described [22]. The conformers above the 1% Boltzmann population were reoptimized. The IEFPCM solvent model was used for TDDFT-ECD calculations via Gaussian 09 at the theoretical B3LYP/6-311G (d, p) level. Finally, the calculated ECD spectrum was obtained and visualized via SpecDis 171 software.

### 4.5. Antibacterial Assay

The fish pathogenic bacterial strains *S. parauberis* KSP28, *P. damselae* FP2244, and *L. garvieae* FP MP5245, were provided by the National Fisheries Research and Development Institute, Korea. *P. aeruginosa* ZJ028 was provided by the Chinese Academy of Tropical Agricultural Sciences. The human pathogenic vancomycin-resistant strains of *E. faecium* G1 were provided by Ruijin Hospital, Shanghai Jiao Tong University School of Medicine. The MIC values for compounds were measured via the 96-well micro-dilution method. Mueller–Hinton II broth (cation-adjusted, BD 212322) was used for bacterial culture. Generally, compounds were dissolved with DMSO to 20 mM as stock solutions. All samples were diluted with culture broth to 2000 µM as the initial concentration. The initial concentration of vancomycin hydrochloride and tetracycline hydrochloride is 172 μM and 0.05 μM, respectively. Further 1:1 serial dilutions were performed by adding culture broth. Next, 10 µL of each drug dilution was distributed in 96-well plates. Then, 190 µL of an exponential-phase bacterial suspension (about 10^5^ CFU/well) was added to each well to reach final concentrations of test compounds ranging from 100 µM to 0.24 µM. Sterile controls were mixed with sterile culture broth only; growth controls were mixed with 10 µL DMSO plus 190 µL bacterial suspension; positive controls were mixed with 10 µL vancomycin hydrochloride or tetracycline hydrochloride plus 190 µL bacterial suspension. The 96-well plates were incubated at 37 °C for 12 h. The MIC values of these compounds were defined as the lowest concentration to completely inhibit the bacterial growth. All MIC values were interpreted according to the recommendations of the Clinical and Laboratory Standards Institute (CLSI) [17].

### 4.6. Cell Viability Assay

The RAW 264.7 cell line was obtained from the National Collection of Authenticated Cell Cultures of China. The cells were cultured in DMEM high-glucose medium supplemented with 10% fetal bovine serum and antibiotics (100 mg/mL streptomycin, 2.5 mg/L amphotericin B) and maintained at 37 °C in a 5% CO_2_ humidified incubator. Cells were seeded into a 96-well plate at a density of 3 × 10^4^ cells per well and cultured overnight. The cells were then treated with test compounds at a final concentration of 50 μM for 24 h. Subsequently, 20 μL of MTT solution (5 mg/mL) was added to each well and incubated at 37 °C in the dark. Afterward, the supernatant was carefully removed, and 150 μL of DMSO was added to each well. Absorbance was measured using a microplate reader at a wavelength of 570 nm.

### 4.7. Production Level of NO in Cell Supernatants

RAW 264.7 cells were seeded into a 96-well plate at a density of 3 × 10^4^ cells per well for 12 h. Cells were pre-treated with compounds or dexamethasone for 1 h at concentrations of 50, 25, 12.5, and 6.25 μM, respectively, and then co-incubated with 50 ng/mL of LPS for 24 h. NO concentrations in cell supernatant were determined using a Griess assay. The Griess reagent (modified) included naphthylethylenediamine dihydrochloride suspended in water and sulfanilamide in phosphoric acid. In accordance with the manufacturer’s instructions, 70 μL of the Griess reagent was mixed with 70 μL of cell supernatant, and then incubated at 37 °C for 15 min. The reagent reacted with nitrite in the samples to form a purple azo product, the absorbance of which was measured at 520 nm. NO concentrations were calculated using 0–100 µM sodium nitrite standards.

### 4.8. Statistical Analysis

The experiments were performed in triplicate and expressed as the mean *±* standard deviation (SD). The data were analyzed by one-way ANOVA. Data were considered statistically significant at *p*-values of <0.05.

## 5. Conclusions

In summary, two new steroids (**1** and **4**), a known natural product (**3**), along with two known steroids (**2** and **5**) were isolated and characterized from South China Sea soft coral *Lobophytum* sp. and sponge *Xestospongia* sp. In the bioassays, compounds **1**, **3**, and **4** exhibited weak antibacterial activities against the vancomycin-resistant *Enterococcus faecium* G1. Compounds **1**, **3**–**5** demonstrated broad-spectrum antibacterial activity against fish pathogenic bacteria. In addition, compound **3** showed dose-dependent anti-inflammatory effects by inhibiting NO production in LPS-stimulated RAW 264.7 cells, exhibiting efficacy comparable to that of dexamethasone. This study highlights the discovery of new polyoxygenated steroids, expanding the chemical diversity and potential pharmacological applications of marine-derived steroids as antibacterial and anti-inflammatory agents. Moreover, the findings emphasize the significant role of the marine environment in shaping the metabolic capacity of invertebrates to produce chemical defense molecules, protecting them from bacterial threats.

## Figures and Tables

**Table 1:** ^1^H and ^13^C NMR data of compounds **1**, **4**, **6,** and **7** in CDCl_3_.

No.	1 ^a^		6 ^b^		4 ^a^		7 ^c^	
	*δ*_C,_ Type	*δ*_H_, Mult (*J* in Hz)	*δ*_C,_ Type	*δ*_H_, Mult (*J* in Hz)	*δ*_C,_ Type	*δ*_H_, Mult (*J* in Hz)	*δ*_C,_ Type	*δ*_H_, Mult (*J* in Hz)
1	32.1, CH_2_	1.77, m	37.1, CH_2_	1.86, m	35.8, CH_2_	2.04, m	37.3, CH_2_	/
		0.91, m		1.09, m		1.72, m		/
2	31.2, CH_2_	1.85, m	31.6, CH_2_	1.84, m	34.1, CH_2_	2.42, m	31.9, CH_2_	/
		1.45, m		1.51, m		2.29, m		/
3	76.6, CH	3.10, m	71.8, CH	3.53, m	199.8, C	-	71.8, CH	/
4	39.4, CH	1.33, m	42.3, CH_2_	2.30, m	123.9, CH	5.72, s	42.3, CH_2_	/
				2.24, m				/
5	51.3, CH	0.77, m	141.0, C	-	171.8, C	-	140.7, C	-
6	24.3, CH_2_	1.72, m	121.5, CH	5.37, d (4.0)	33.1, CH_2_	2.40, m	121.7, CH	5.35, d (5.4)
		1.09, m				2.30, m		
7	36.8, CH_2_	1.74, m	31.5, CH_2_	2.01, m	32.2, CH_2_	1.86, m	31.6, CH_2_	/
		1.05, m		1.62, m		1.04, m		/
8	33.6, CH	1.56, m	30.4, CH	1.69, m	36.3, CH	1.44, m	36.4, CH	/
9	54.9, CH	0.71, m	50.3, CH	1.02, m	53.9, CH	0.93, m	50.1, CH	/
10	36.3, C	-	36.6, C	-	38.8, C	-	36.5, C	-
11	21.3, CH_2_	1.61, m	20.9, CH_2_	1.60, m	21.2, CH_2_	1.55, m	21.1, CH_2_	/
		1.39, m		1.60, m		1.45, m		/
12	36.5, CH_2_	2.06, m	36.2, CH_2_	2.10, m	39.7, CH_2_	2.05, m	39.7, CH_2_	/
		1.56, m		1.59, m		1.19, m		
13	47.7, C	-	47.4, C	-	42.6, C	-	42.3, C	-
14	58.1, CH	1.39, m	57.9, CH	1.41, m	56.0, CH	1.05, m	56.7, CH	/
15	31.1, CH_2_	2.08, m	31.0, CH_2_	2.06, m	24.4, CH_2_	1.64, m	24.3, CH_2_	/
		1.46, m		1.87, m		1.14, m		/
16	124.0, CH	5.49, br d (1.8)	123.8, CH	5.50, d (1.5)	28.4, CH_2_	1.86, m	28.3, CH_2_	/
						1.30, m		/
17	161.1, C	-	160.9, C	-	55.7, CH	1.15, m	55.6, CH	/
18	18.4, CH_3_	0.98, s	18.1, CH_3_	1.00, s	12.1, CH_3_	0.72, s	11.8, CH_3_	0.69, s
19	13.5, CH_3_	0.88, s	19.3, CH_3_	1.04, s	17.5, CH_3_	1.18, s	19.4, CH_3_	1.01, s
20	76.0, C	-	75.9, C	-	36.5, CH	1.15, m	34.7, CH	/
21	29.7, CH_3_	1.36, s	29.6, CH_3_	1.37, s	18.8, CH_3_	0.98, d (6.6)	18.7, CH_3_	1.01, d (6.9)
22	49.3, CH_2_	1.61, m	49.0, CH_2_	1.57, m	35.8, CH_2_	2.04, m	29.7, CH_2_	/
		1.51, m		1.50, m		1.72, m		/
23	29.8, CH	1.28, m	29.6, CH	1.83, m	26.7, CH_2_	2.15, td (12.6, 4.6)	26.6, CH_2_	/
						1.95, td (12.5, 5.1)		/
24	45.6, CH	1.08, m	45.4, CH	1.07, m	155.4, C	-	155.2, C	-
25	31.0, CH	1.45, m	30.8, CH	1.42, m	34.8, CH	2.27, m	31.9, CH	/
26	21.6, CH_3_	0.91, d (6.6)	21.4, CH_3_	0.89, d (7.0)	22.1, CH_3_	1.03, d (6.8)	21.9, CH_3_	1.03, d (6.9)
27	21.1, CH_3_	0.89, d (5.8)	21.0, CH_3_	0.86, d (7.0)	22.2, CH_3_	1.04, d (6.8)	22.0, CH_3_	1.03, d (6.9)
28	11.7, CH_3_	0.78, d (6.8)	11.6, CH_3_	0.76, d (7.0)	115.1, CH	5.32, t (7.3)	114.9, CH	5.31, t (6.9)
29	15.8, CH_3_	0.80, d (6.9)	15.7, CH_3_	0.78, d (7.0)	73.6, CH_2_	4.55, d (7.3)	73.4, CH_2_	4.56, d (6.9)
						4.55, d (7.3)		4.56, d (6.9)
30	15.3, CH_3_	0.97, d (5.6)						
OOH						7.88, s		8.04, br s

^a^ Recorded in CDCl_3_ at 600 MHz for ^1^H and 150 MHz for ^13^C NMR. s (singlet), d (doublet), t (triplet), m (multiplet), td (triplet of doublet), and br s (broad singlet). ^b^ Recorded in ref. [12]. ^c^ Recorded in ref. [13], “/” indicates not assigned.

**Table 2:** The MIC values (μM) of antimicrobial activity of compounds **1** and **3**–**5**.

Compounds	1	3	4	5	TC	VA
(VRE) *E. faecium* G1	100	100	100	-	0.025	>172
*S. parauberis* FP KSP28	100	100	-	-	0.006	NT
*P. damselae* FP2244	50	100	-	-	0.00004	NT
*P. aeruginosa* ZJ028	-	-	-	100	0.025	NT
*L. garvieae* FP MP5245	50	100	100	-	0.0008	NT

VRE: vancomycin-resistant enterococcus; TC: tetracycline; VA: vancomycin; “-”: the result was negative at the concentration of 100 μΜ; NT: not tested.

## Data Availability

The data that support the findings of this study are available in the Supplementary Material of this article.

## Supplementary Materials

The following supporting information can be downloaded at https://www.mdpi.com/article/10.3390/md23010036/s1: Figures S1–S15: NMR, HR-ESIMS, and IR data of compounds **1** and **4**; Figure S16: UV and ECD spectra of compound **4**; Figure S17: Structure of isomer (8*S*,9*S*,10*R*,13*R*,14*S*,17*R*,20*R*)-**4** studied for TDDFT-ECD calculation; Figures S18–S21, S23, and S24: ^1^H and ^13^C NMR data of compounds **2**, **3**, and **5**; and Figure S22: UV and ECD spectra of compound **3**.

## References

[1] Rodrigues I.G., Miguel M.G., Mnif W. (2019). A brief review on new naturally occurring cembranoid diterpene derivatives from the soft corals of the genera *Sarcophyton*, *Sinularia*, and *Lobophytum* since 2016. Molecules.

[2] Zhou X., Xu T., Yang X.W., Huang R., Yang B., Tang L., Liu Y. (2010). Chemical and biological aspects of marine sponges of the genus Xestospongia. Chem. Biodivers..

[3] Savić M.P., Sakač M.N., Kuzminac I.Z., Ajduković J.J., Biology M. (2022). Structural diversity of bioactive steroid compounds isolated from soft corals in the period 2015–2020. J. Steroid Biochem..

[4] Ke S. (2018). Recent progress of novel steroid derivatives and their potential biological properties. Mini-Rev. Med. Chem..

[5] Tran H.H.T., Viet P.N., Van T.N., Tran H.T., Xuan C.N., Hoai N.N., Cong T.D., Van K.P., Van M.C. (2017). Cytotoxic steroid derivatives from the Vietnamese soft coral *Sinularia brassica*. J. Asian Nat. Prod. Res..

[6] Tammam M.A., Rarova L., Kvasnicova M., Gonzalez G., Emam A.M., Mahdy A., Strnad M., Ioannou E., Roussis V. (2020). Bioactive steroids from the Red Sea soft coral *Sinularia polydactyla*. Mar. Drugs.

[7] Liu J., Wu X., Yang M., Gu Y.-C., Yao L.-G., Huan X.-J., Miao Z.-H., Luo H., Guo Y.-W. (2020). Erectsterates A and B, a pair of novel highly degraded steroid derivatives from the South China Sea soft coral *Sinularia erecta*. Steroids.

[8] Yan X., Liu J., Leng X., Ouyang H. (2021). Chemical diversity and biological activity of secondary metabolites from soft coral genus *Sinularia* since 2013. Mar. Drugs.

[9] Kokke W., Bohlin L., Fenical W., Djerassi C. (1982). Novel dinoflagellate 4*α*-methylated sterols from four caribbean gorgonians. Phytochemistry.

[10] Boto A., Freire R., Hernández R., Suárez E., Rodríguez M.S. (1997). Tandem β-fragmentation-hydrogen abstraction reaction of alkoxy radicals in steroidal systems. J. Org. Chem..

[11] Zhang X.W., Tang X.L., Liu B.S., Li P.L., Li G.Q. (2016). Biodiversity, Characteristic steroids from the South China Sea gorgonian *Muricella sibogae* and their cytotoxicities. Chem. Biodivers..

[12] Lu Y., Lin Y.-C., Wen Z.-H., Su J.-H., Sung P.-J., Hsu C.-H., Kuo Y.-H., Chiang M.Y., Dai C.-F., Sheu J.-H. (2010). Steroid and cembranoids from the Dongsha atoll soft coral *Lobophytum sarcophytoides*. Tetrahedron.

[13] Sheu J.-H., Wang G.-H., Sung P.-J., Chiu Y.-H., Duh C.-Y. (1997). Cytotoxic sterols from the formosan brown alga *Turbinaria ornata*. Planta Medica.

[14] Huang C.-Y., Tseng W.-R., Ahmed A.F., Chiang P.-L., Tai C.-J., Hwang T.-L., Dai C.-F., Sheu J.-H. (2018). Anti-inflammatory polyoxygenated steroids from the soft coral *Lobophytum michaelae*. Mar. Drugs.

[15] Zhang D., Wang Z., Han X., Li X.-L., Lu Z.-Y., Dou B.-B., Zhang W.-Z., Tang X.-L., Li P.-L., Li G.-Q. (2022). Four bioactive new steroids from the soft coral *Lobophytum pauciflorum* collected in South China Sea. Beilstein J. Org. Chem..

[16] Yang M., Cui W.-X., Li H., Li S.-W., Yao L.-G., Tang W., Mudianta I.W., Guo Y.-W. (2020). Sinulasterols A–C, three new bioactive oxygenated steroids from the South China Sea soft coral *Sinularia depressa*. Steroids.

[17] Zhu S.-H., Chang Y.-M., Su M.-Z., Yao L.-G., Li S.-W., Wang H., Guo Y.-W. (2024). Nine new antibacterial diterpenes and steroids from the South China Sea soft coral *Lobophytum catalai* tixier-durivault. Mar. Drugs.

[18] Xu T., Zhao Q.-M., Yao L.-G., Lan L.-F., Li S.-W., Guo Y.-W. (2023). Sinulasterols D–G, four new antibacterial steroids from the South China sea soft coral *Sinularia depressa*. Steroids.

[19] Xia Z.-Y., Sun M.-M., Jin Y., Yao L.-G., Su M.-Z., Liang L.-F., Wang H., Guo Y.-W. (2023). Lobosteroids A–F: Six New Highly Oxidized Steroids from the Chinese Soft Coral *Lobophytum* sp.. Mar. Drugs.

[20] Ralambondrahety R., Couzinet-Mossion A., Rabesaotra V., Andriambeloson O., Barnathan G., Rakotovao M., Wielgosz-Collin G. (2021). Antibacterial activity of steroids isolated from the madagascar marine sponge biemna laboutei: Δ7 steroids as new potential agents against pathogenic bacteria. Nat. Prod. J..

[21] Wen H.-M., Zhang Y.-W., Feng F.-J., Huang G.-B., Lv Y.-H., Zhang Z.-Y., Ding L.-J. (2024). Antibacterial oxygenated ergostane-type steroids produced by the marine sponge-derived fungus *Aspergillus* sp.. J. Asian Nat. Prod. Res..

[22] Pescitelli G., Bruhn T. (2016). Good computational practice in the assignment of absolute configurations by TDDFT calculations of ECD spectra. Chirality.

