# Supplementary material for: Bioactive Steroids with Structural Diversity from the South China Sea Soft Coral Lobophytum sp. and Sponge Xestospongia sp
Source: Mar Drugs. 2025 Jan 13;23(1):36. doi: 10.3390/md23010036 (PMC11766953; doi:10.3390/md23010036)

Bioactive Steroids with Structural Diversity from the South China Sea soft coral *Lobophytum* sp. and sponge *Xestospongia* sp.

Lin-Mao Ke[1,†], Zi-Ru Zhang[1,2,3,†], Song-Wei Li[4], Yan-Bo Zeng[2,*], Ming-Zhi Su[1,*], Yue-Wei Guo[1,4,*]

[1] *Shandong Laboratory of Yantai Drug Discovery , Bohai Rim Advanced Research Institute for Drug Discovery, Yantai，Shandong 264117, China;* klm102198@163.com (L.-M.K.)

[2] *Hainan Provincial Key Laboratory for Functional Components Research and Utilization of Marine Bio-resources, Institute of Tropical Bioscience and Biotechnology, Chinese Academy of Tropical Agricultural Sciences, Haikou 571101, China;* zengyanbo@itbb.org.cn (Y.-B.Z.)

[3] *Ocean College of Hebei Agricultural University, Qinhuangdao 066000, China;* 18789783359@139. com (Z.-R.Z.)

[4] *School of Medicine, Shanghai University, Shanghai 200444, China;* songweili@shu.edu.cn (S.-W.L.)

* Corresponding author

E-mail address: ywguo@simm.ac.cn (Y.-W.G.); mzsu@baridd.ac.cn (M.-Z.S.); zengyanbo@itbb.org.cn (Y.-B.Z.)

[†]These authors contributed equally to this work.

Content

Original spectra of 1 3

Figure S1. ^{1}H (600 MHz) NMR spectrum of compound 1 in $CDCl_3$. 3

Figure S2. ^{13}C (150 MHz) NMR spectrum of compound 1 in $CDCl_3$. 3

Figure S3. HSQC (600 MHz) spectrum of compound 1 in $CDCl_3$ 4

Figure S4. HMBC (600 MHz) spectrum of compound 1 in $CDCl_3$. 4

Figure S5. ^{1}H–^{1}H COSY (600 MHz) spectrum of compound 1 in $CDCl_3$. 5

Figure S6. NOESY (600 MHz) spectrum of compound 1 in $CDCl_3$. 5

Figure S7. HR-ESIMS spectrum of compound 1. 6

Figure S8. IR spectrum of compound 1. 7

Original spectra of 4 7

Figure S9. ^{1}H (600 MHz) NMR spectrum of compound 4 in $CDCl_3$. 7

Figure S10. ^{13}C (150 MHz) NMR spectrum of compound 4 in $CDCl_3$. 8

Figure S11. HSQC (600 MHz) spectrum of compound 4 in $CDCl_3$. 8

Figure S12. HMBC (600 MHz) spectrum of compound 4 in $CDCl_3$. 9

Figure S13. ^{1}H–^{1}H COSY (600 MHz) spectrum of compound 4 in $CDCl_3$. 9

Figure S14. HR-ESIMS spectrum of compound 4. 10

Figure S15. IR spectrum of compound 4. 11

Figure S16. UV and ECD spectra of compound 4. 11

Computational details (TDDFT-ECD) of 4 11

Figure S17. Structure of isomer (8*S*,9*S*,10*R*,13*R*,14*S*,17*R*,20*R*)-4 studied for TDDFT-ECD calculation. 11

Original spectra of known compounds 2, 3, and 5 12

Figure S18. ^{1}H (600 MHz) NMR spectrum of compound 2 in $CDCl_3$. 12

Figure S19. ^{13}C (150 MHz) NMR spectrum of compound 2 in $CDCl_3$. 12

Figure S20. ^{1}H (600 MHz) NMR spectrum of compound 3 in $CDCl_3$. 13

Figure S21. ^{13}C (150 MHz) NMR spectrum of compound 3 in $CDCl_3$. 13

Figure S22. UV and ECD spectra of compound 3. 14

Figure S23. ^{1}H (600 MHz) NMR spectrum of compound 5 in CDCl 14

Figure S24. ^{13}C (150 MHz) NMR spectrum of compound 5 in $CDCl_3$. 15

Original spectra of 1

Figure S1. ^{1}H (600 MHz) NMR spectrum of compound **1** in $CDCl_3$.

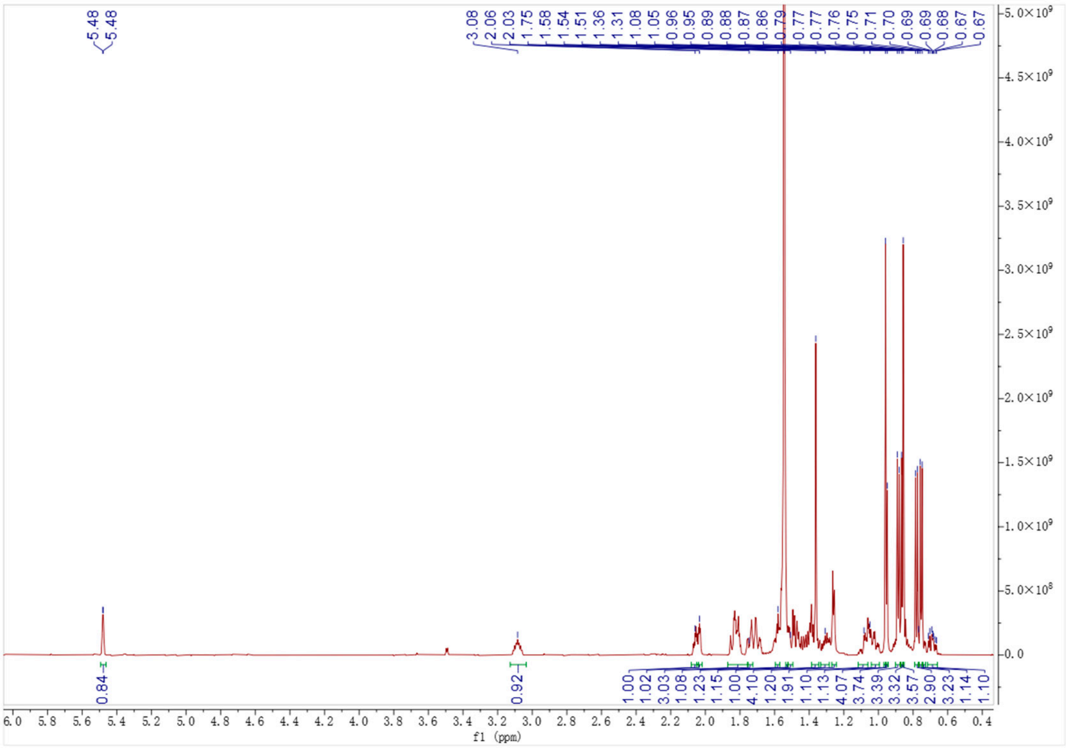

Figure S2. ^{13}C (150 MHz) NMR spectrum of compound **1** in $CDCl_3$.

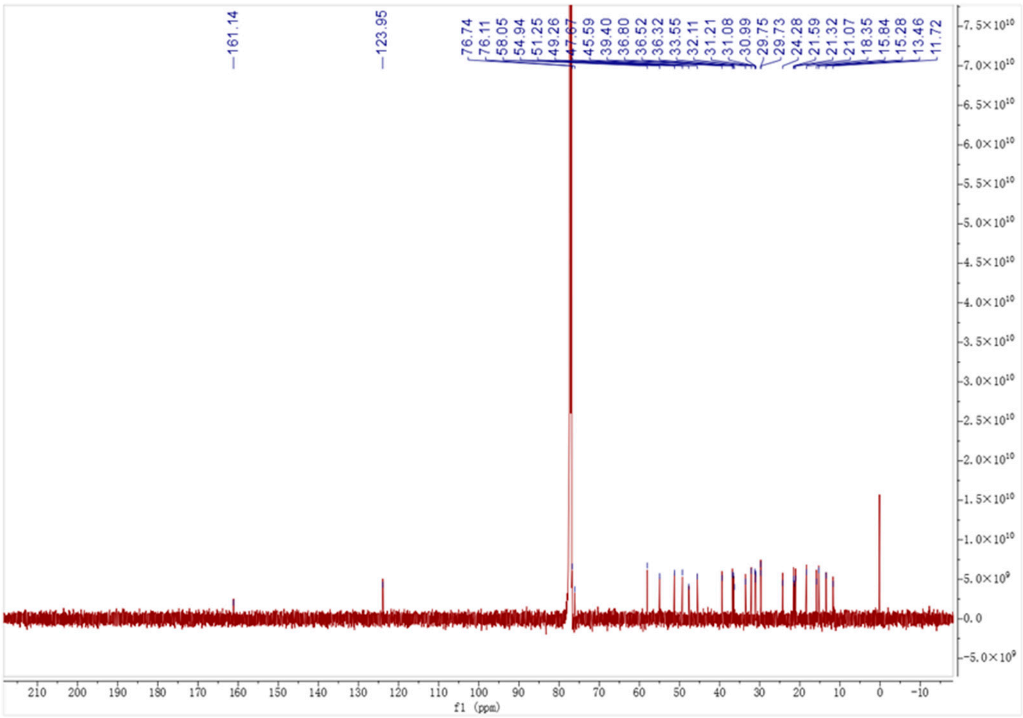

Figure S3. HSQC (600 MHz) spectrum of compound **1** in $CDCl_3$

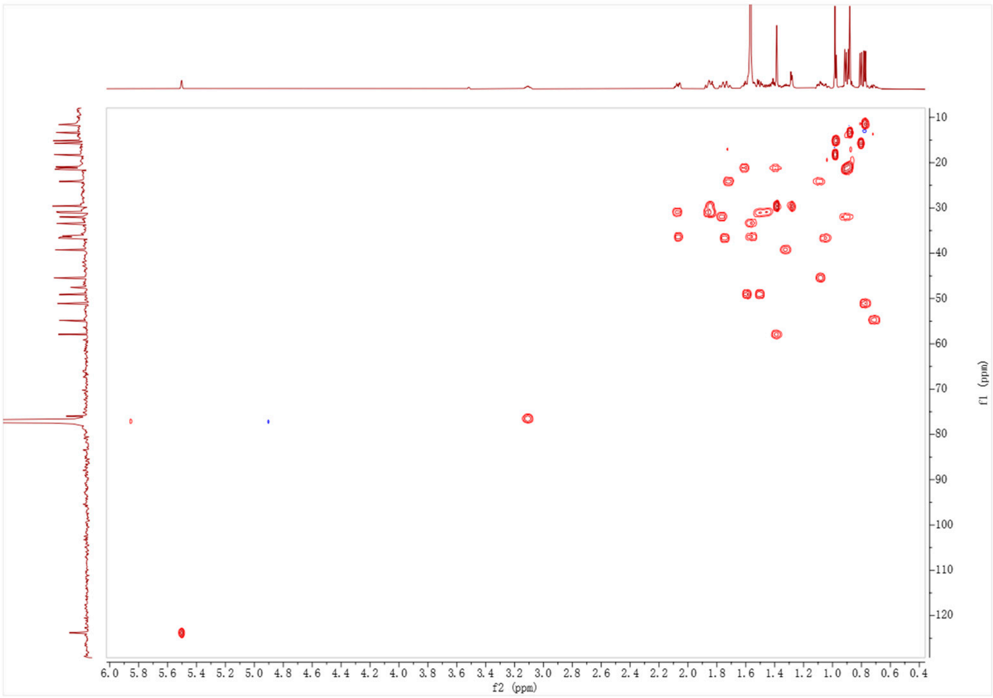

Figure S4. HMBC (600 MHz) spectrum of compound **1** in $CDCl_3$.

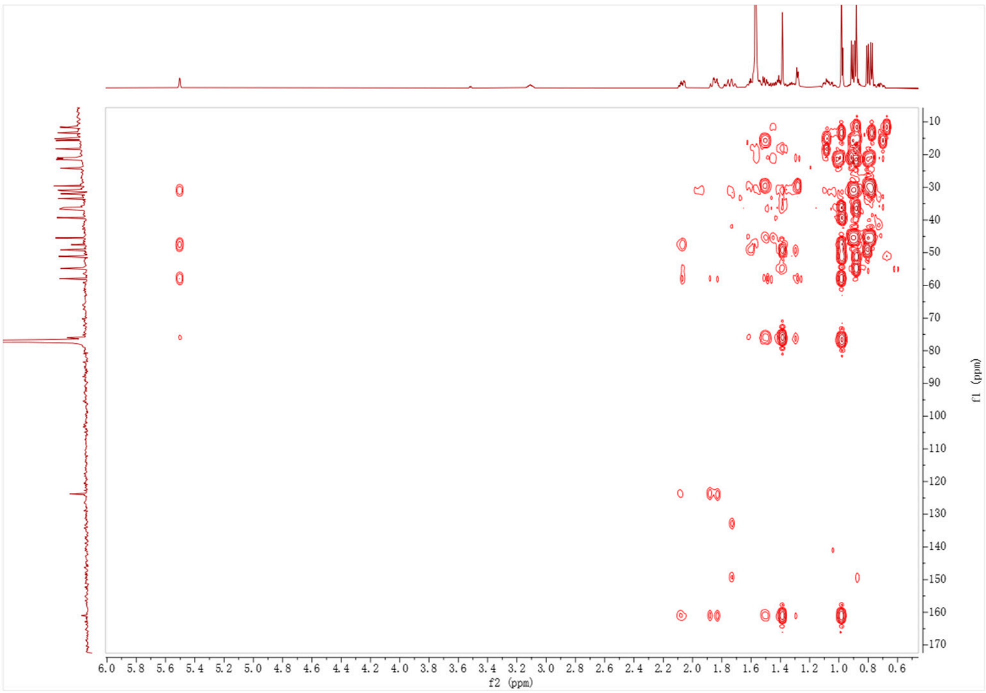

Figure S5. ^{1}H–^{1}H COSY (600 MHz) spectrum of compound **1** in $CDCl_3$.

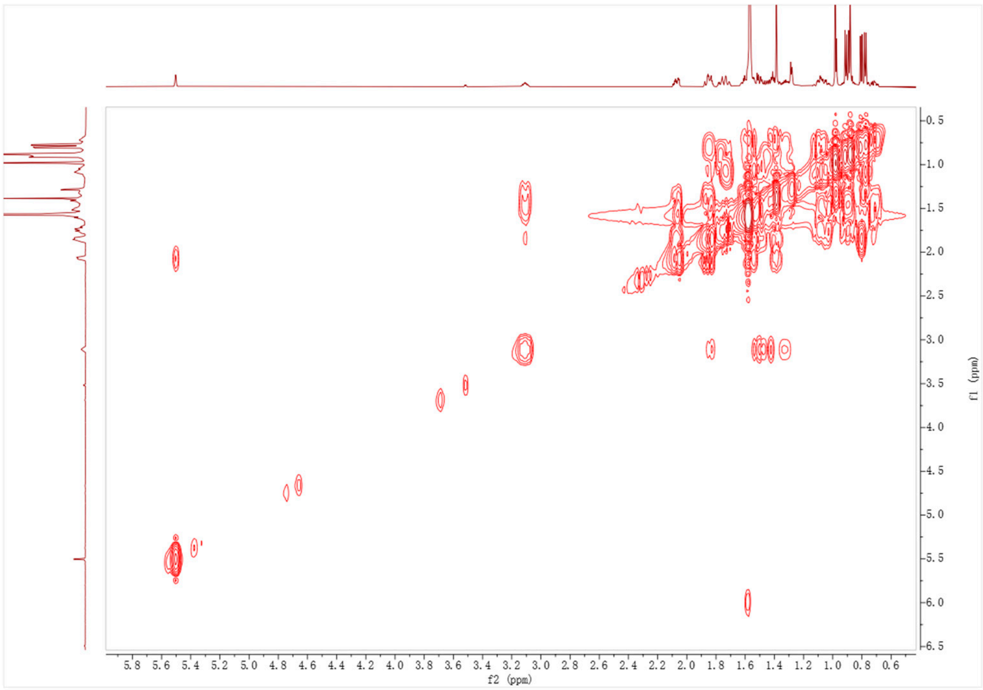

Figure S6. NOESY (600 MHz) spectrum of compound **1** in $CDCl_3$.

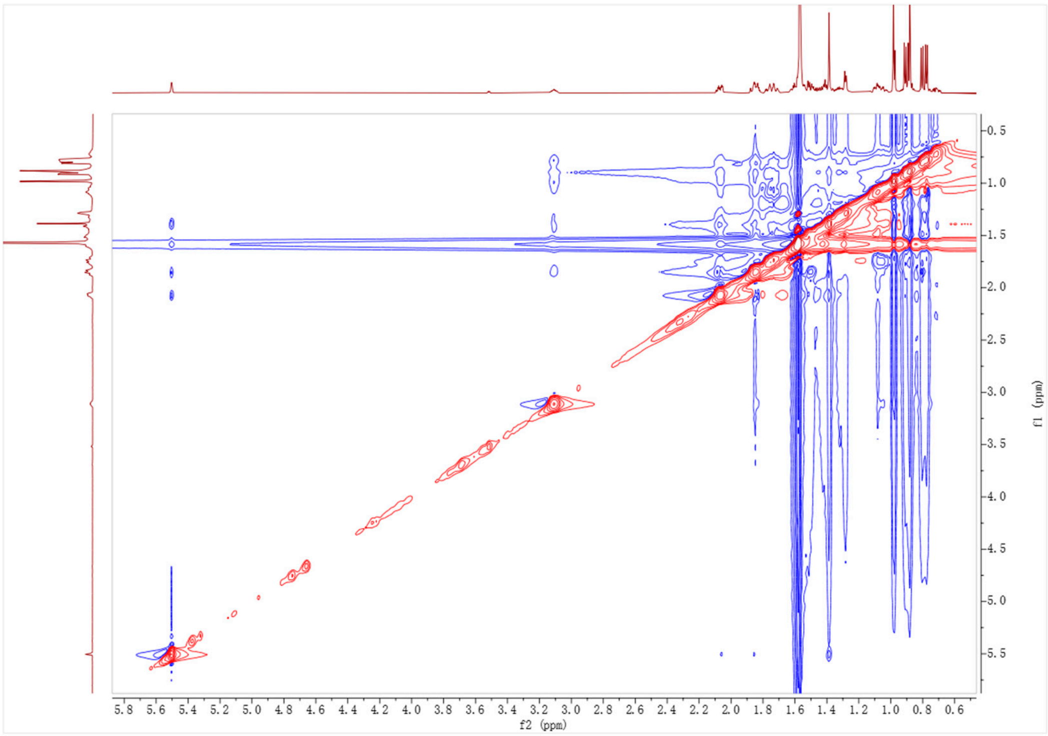

Figure S7. HR-ESIMS spectrum of compound **1**.

Qualitative Analysis Report

SCIEX

2024-05-08

Data File: ZR-1	Sample Name: ZR-1
Instrument Name: Sciex ZenoTOF™ 7600 System	Polarity: Positive
LC Method: LC-5min.lcm	MS Method: MS1-Pos-100-2000.msm
Calibration: Pass	

MS spectra

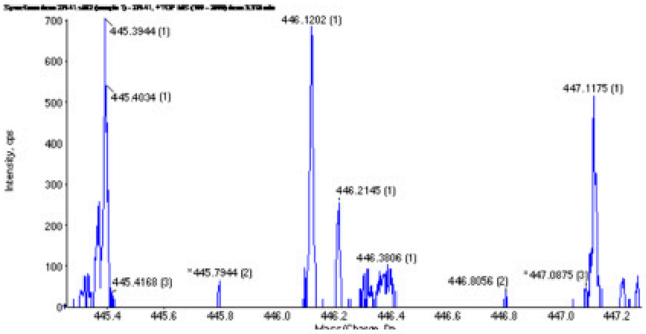

Formula Calculator Results

Measure m/z	Cal m/z	Error(mmu)	Error(ppm)	Ion Formula	Ion
445.4034	445.4040	-0.6	-1.4	C30H53O2	[M+H]+

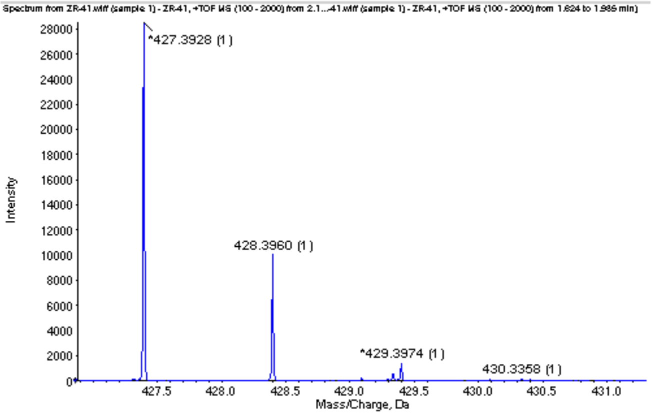

Formula Calculator Results

Measure m/z	Cal m/z	Error(mmu)	Error(ppm)	Ion Formula	Ion
427.3928	427.3934	-0.6	-1.5	C30H51O	[M-H2O+H]+

Figure S8. IR spectrum of compound **1**.

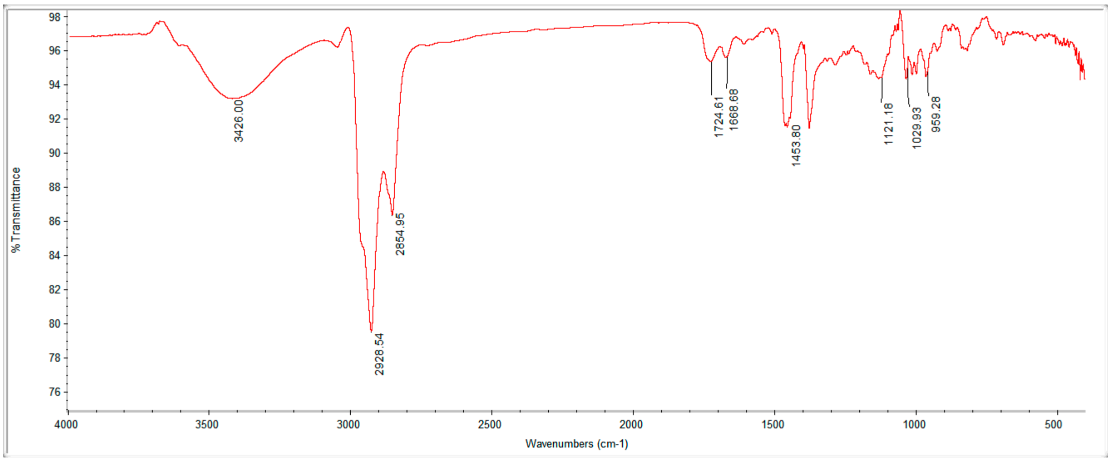

Original spectra of 4

Figure S9. ^{1}H (600 MHz) NMR spectrum of compound **4** in $CDCl_3$.

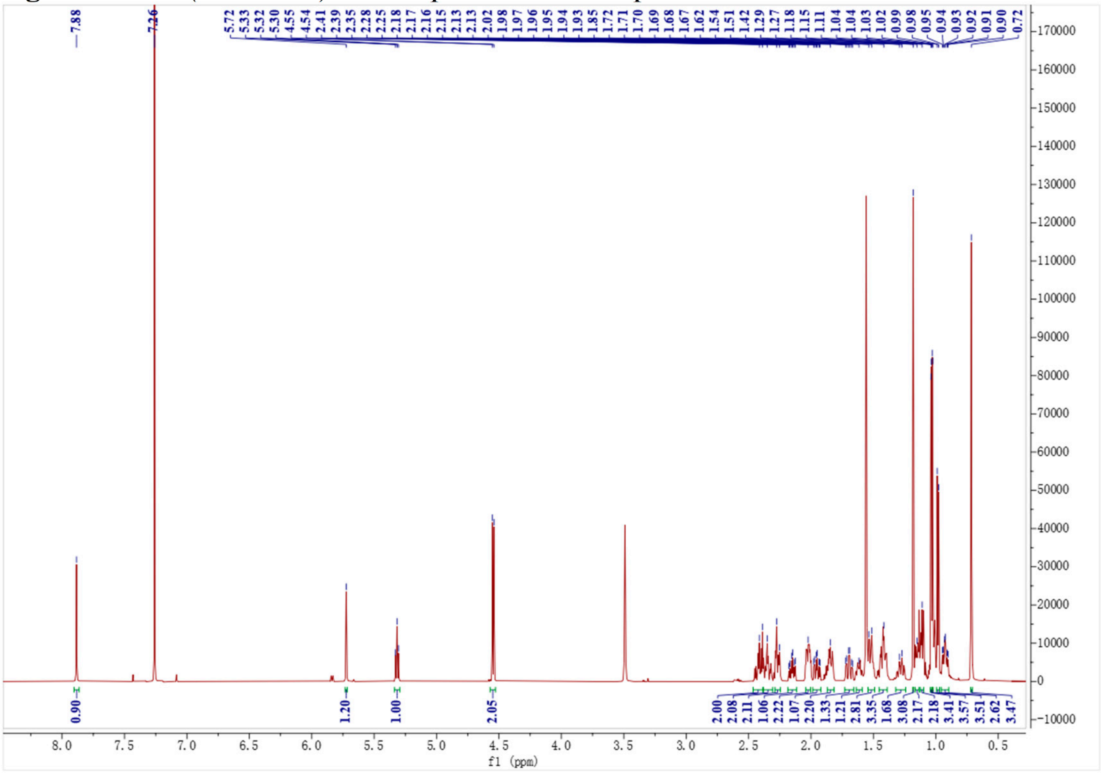

Figure S10. ^{13}C (150 MHz) NMR spectrum of compound **4** in $CDCl_3$.

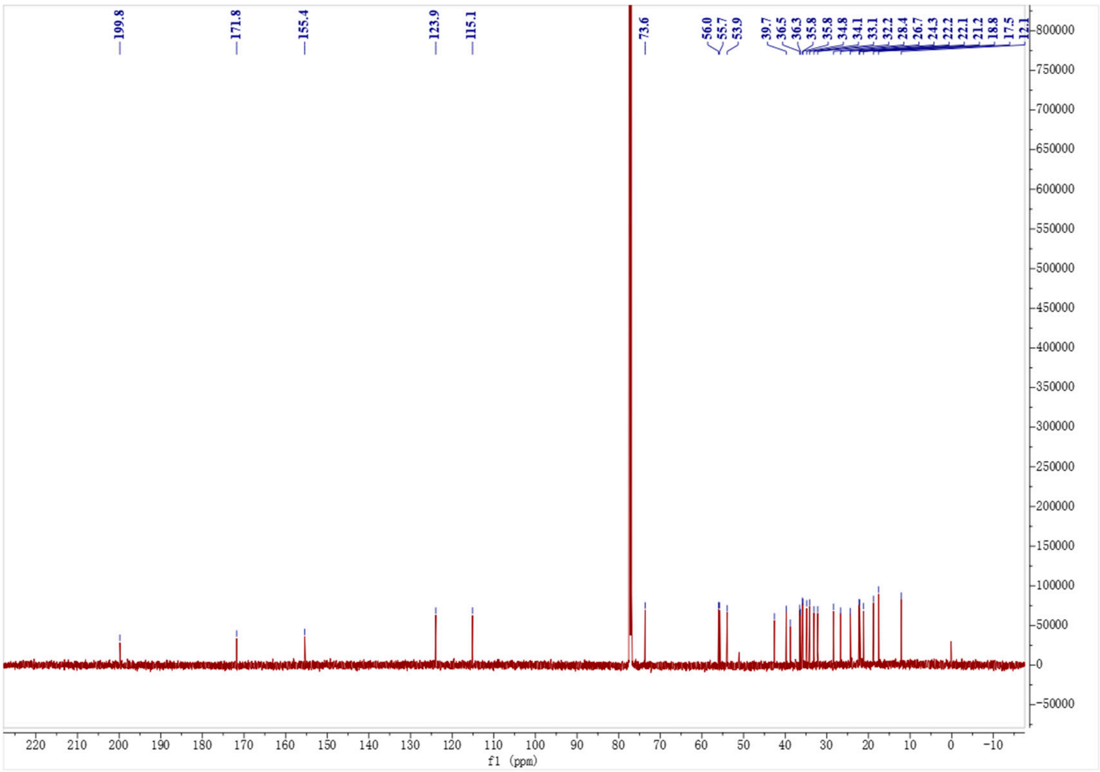

Figure S11. HSQC (600 MHz) spectrum of compound **4** in $CDCl_3$.

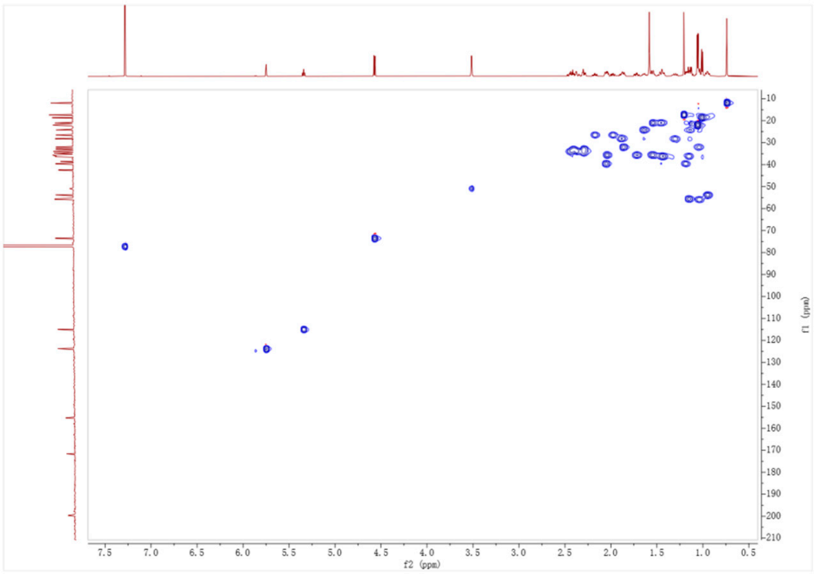

Figure S12. HMBC (600 MHz) spectrum of compound **4** in $CDCl_3$.

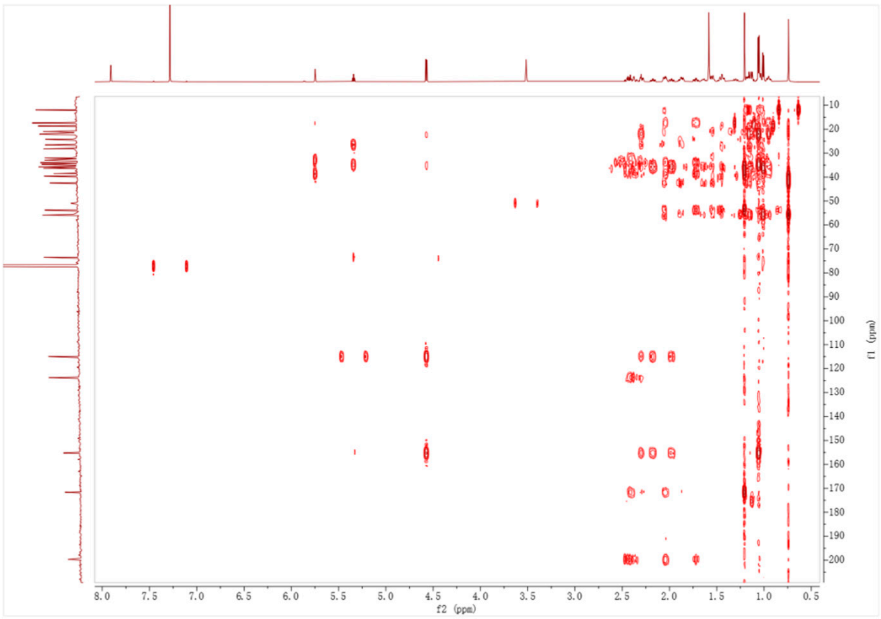

Figure S13. ^1H–^1H COSY (600 MHz) spectrum of compound **4** in $CDCl_3$.

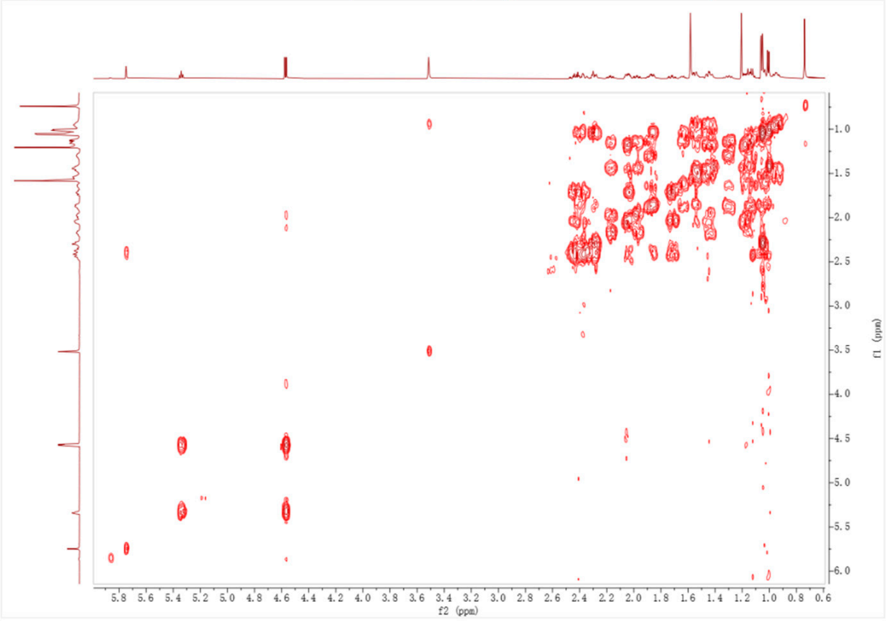

Figure S14. HR-ESIMS spectrum of compound **4**.

MS spectra

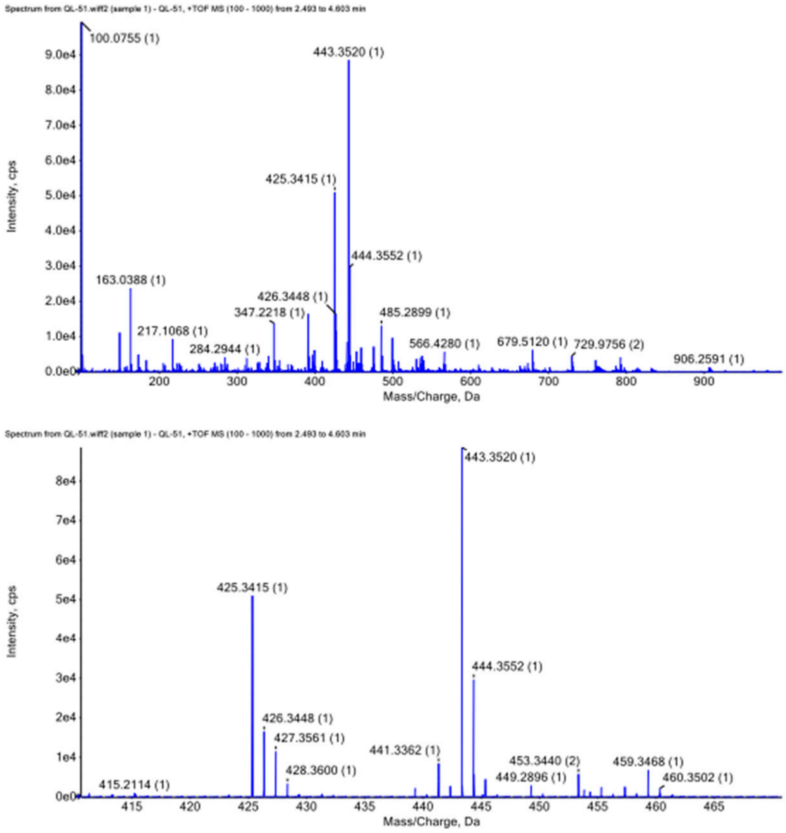

Formula Calculator Results

Measured m/z	Cal m/z	Error(mmu)	Error(ppm)	Ion Formula	Ion
443.3520	443.3520	0	0	C29H47O3	$[M+H]^+$
425.3415	425.3414	0.1	0.2	C29H45O2	$[M+H-H_2O]^+$

Figure S15. IR spectrum of compound **4**.

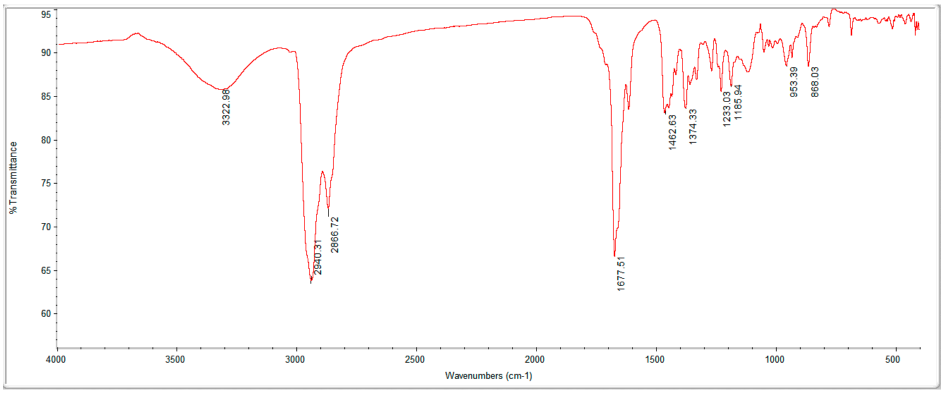

Figure S16. UV and ECD spectra of compound **4**.

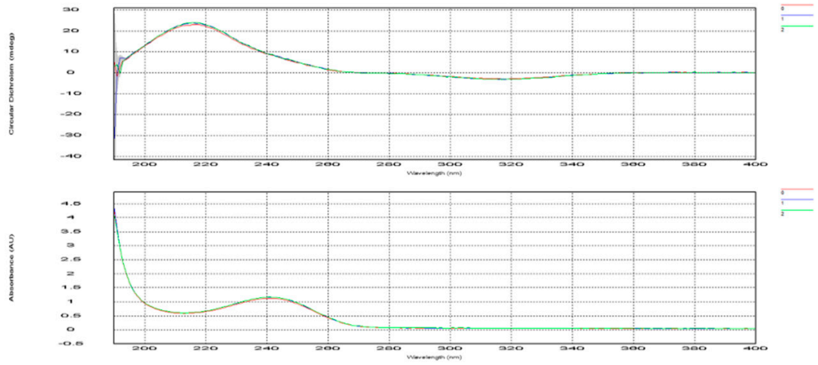

Computational details (TDDFT-ECD) of 4

Figure S17. Structure of isomer (8*S*,9*S*,10*R*,13*R*,14*S*,17*R*,20*R*)-**4** studied for TDDFT-ECD calculation.

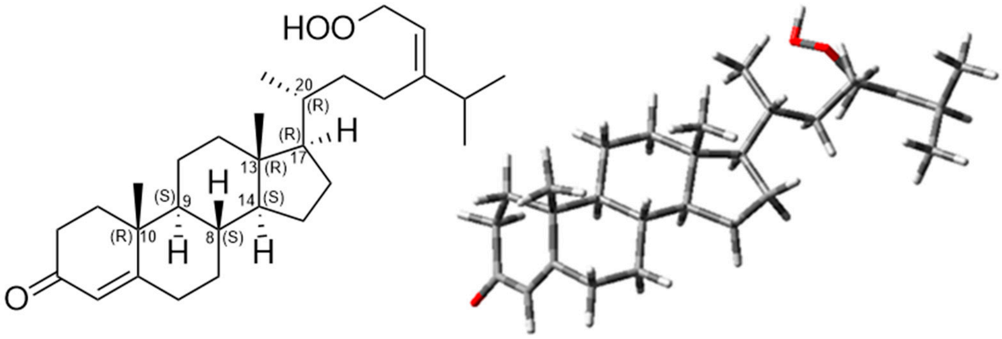

Original spectra of known compounds 2, 3, and 5

Figure S18. ^{1}H (600 MHz) NMR spectrum of compound **2** in $CDCl_3$.

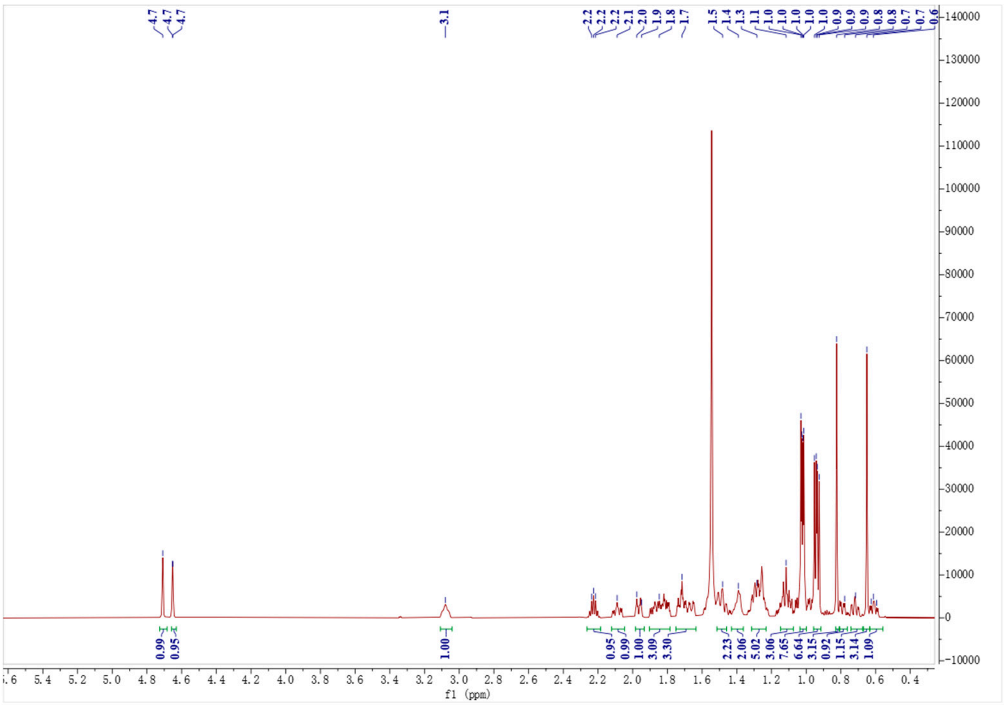

Figure S19. ^{13}C (150 MHz) NMR spectrum of compound **2** in $CDCl_3$.

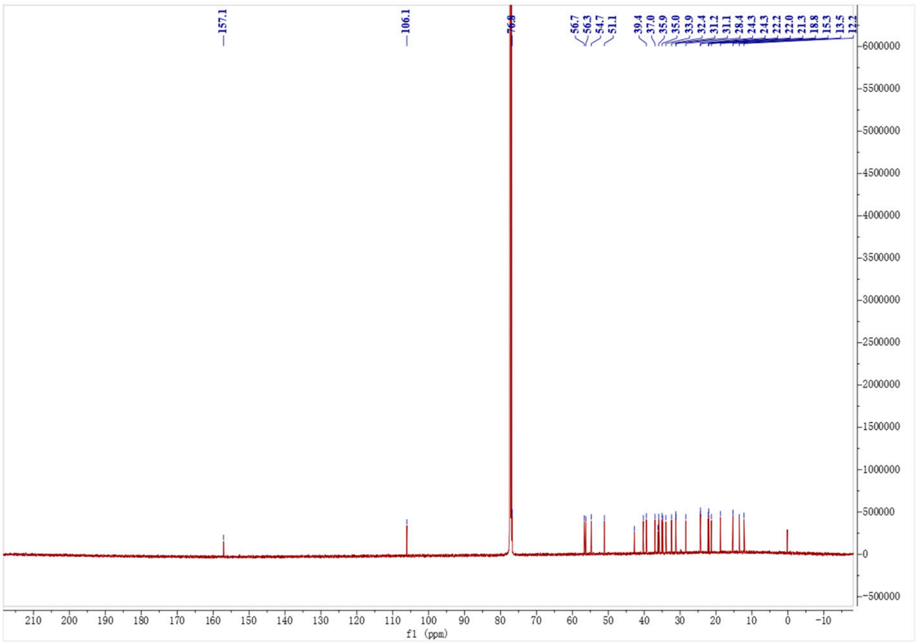

Figure S20. ^{1}H (600 MHz) NMR spectrum of compound **3** in $CDCl_3$.

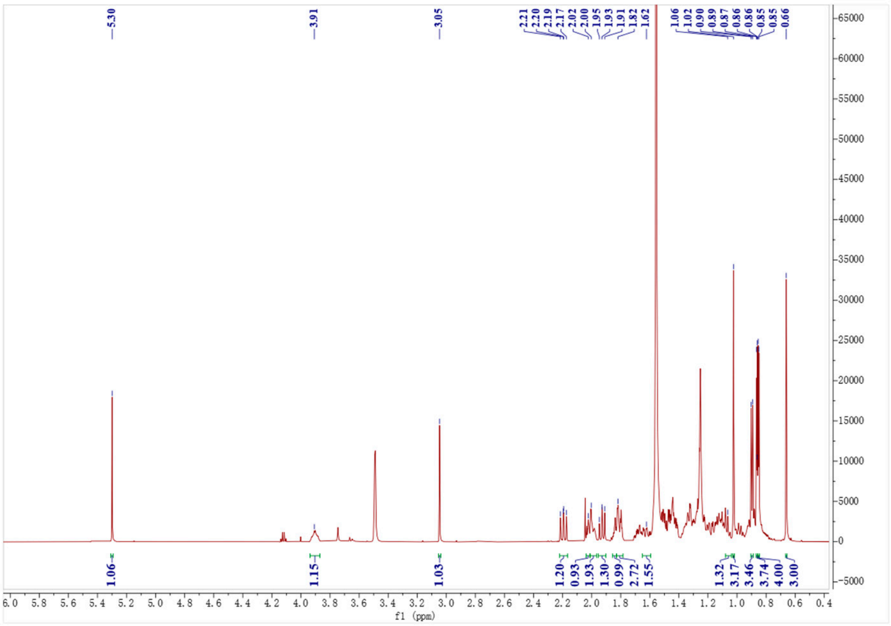

Figure S21. ^{13}C (150 MHz) NMR spectrum of compound **3** in $CDCl_3$.

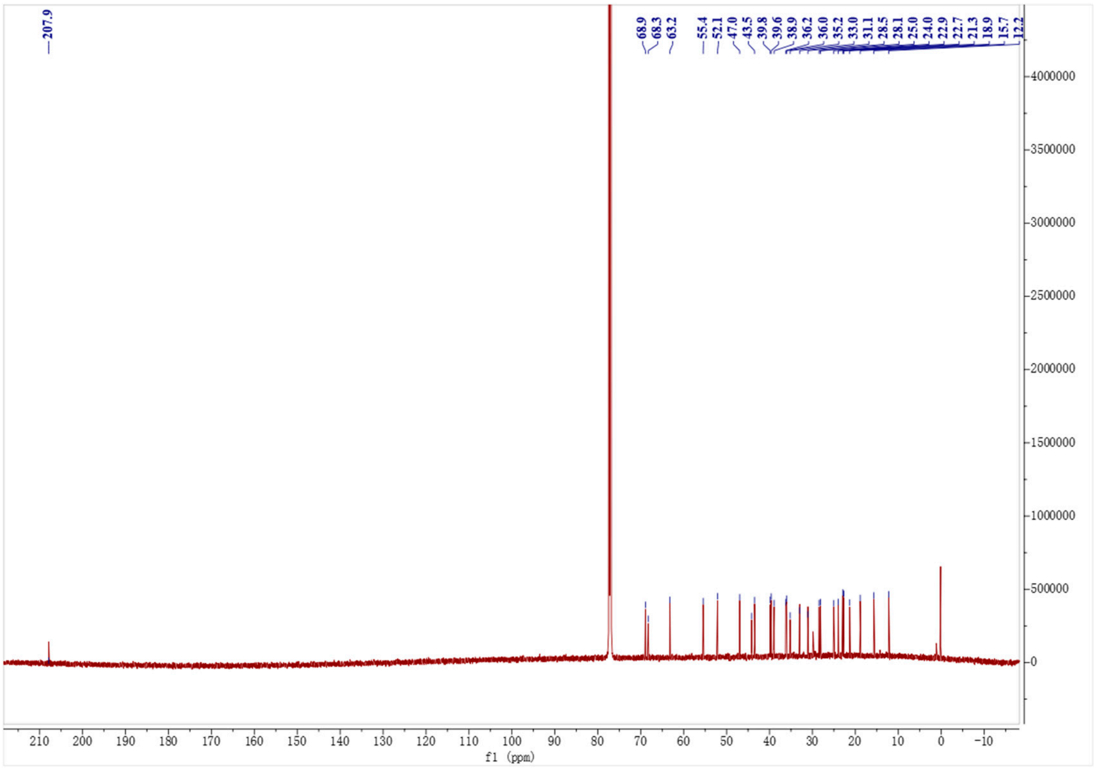

Figure S22. UV and ECD spectra of compound **3**.

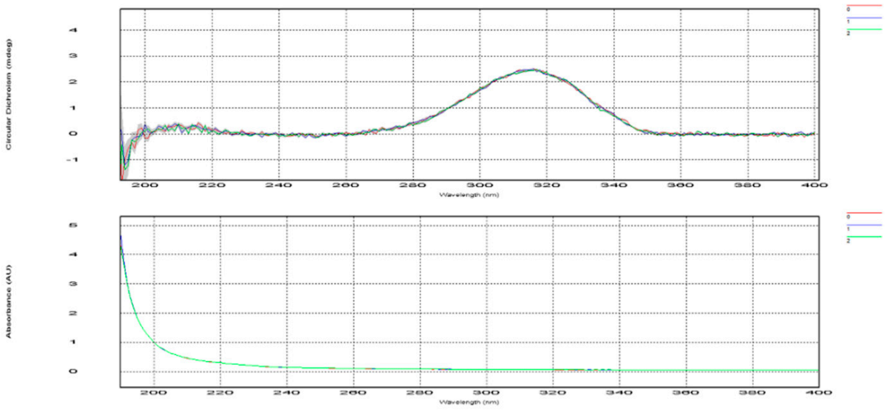

Figure S23. ^{1}H (600 MHz) NMR spectrum of compound **5** in CDCl

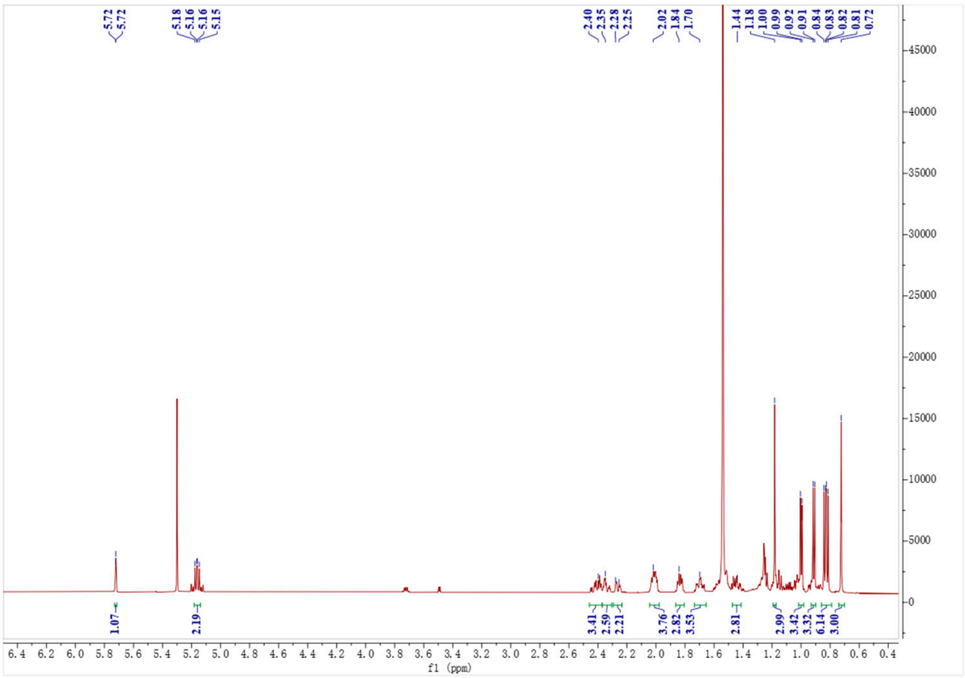

Figure S24. ^{13}C (150 MHz) NMR spectrum of compound **5** in $CDCl_3$.

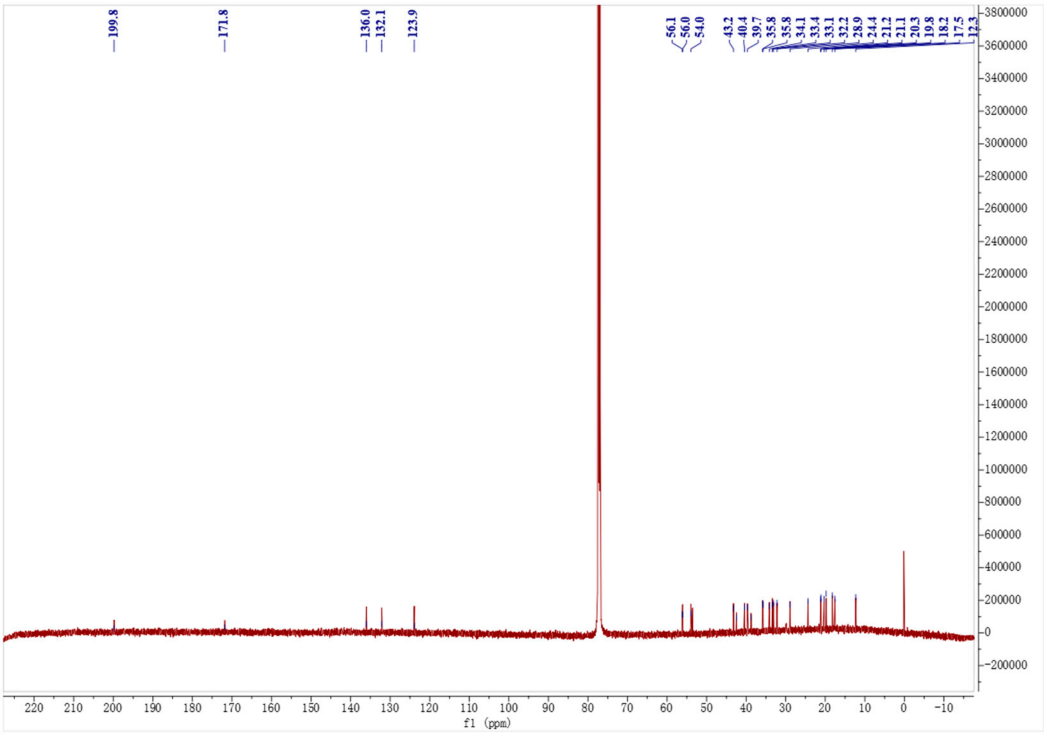

Supplement: Supplementary file 1 [file marinedrugs-23-00036-s001.zip › marinedrugs-3392403-supplementary.pdf]
